# Correction: Nuclei Segmentation and Classification from Histopathology Images using Federated Learning for End-Edge Platform

**DOI:** 10.1371/journal.pone.0339426

**Published:** 2025-12-29

**Authors:** The PLOS One Staff

## Notice of Republication

This article was republished on December 3, 2025, to correct the figures which were out of order. The order has now been corrected. Please download this article again to view the correct version. The originally published, uncorrected article and the republished, corrected articles are provided here for reference.

## Supporting information

S1 File Originally published, uncorrected article. (PDF)

S2 File Republished, corrected article. (PDF)
