# Expression of Concern: Nuclei segmentation and classification from histopathology images using federated learning for end-edge platform

**DOI:** 10.1371/journal.pone.0361682

**Published:** 2026-10-30

**Authors:** The PLOS One Editors

Contrary to the Data Availability statement in [1,2], the individual-level underlying data supporting the article’s results were not provided with the article.

The authors provided information on third-party datasets used in this article (provided here: https://www.kaggle.com/datasets/theredlad/pannuke-dataset-experimental-data/data). However, they did not provide the data underlying the figures and graphs on request, which the Editors consider to be necessary for compliance with the PLOS Data Availability policy. Therefore, the *PLOS One* Editors issue this Expression of Concern.

In addition, the authors were unable to provide the source code for [1].

The authors have supplied recreated code, which they have made publicly available:

- Code repository: https://github.com/anzir29/pannuke-nuclei-fl
- Archived release (v1.0): https://doi.org/10.5281/zenodo.23142912

We regret that the issues were not addressed prior to the article’s publication.
