# Supplementary material for: Correction: Nuclei Segmentation and Classification from Histopathology Images using Federated Learning for End-Edge Platform
Source: PLoS One. 2025 Dec 29;20(12):e0339426. doi: 10.1371/journal.pone.0339426 (PMC12747367; doi:10.1371/journal.pone.0339426)
Supplement: S1 File — (PDF) [file pone.0339426.s001.pdf]

PLOS ONE

RESEARCH ARTICLE

Nuclei segmentation and classification from histopathology images using federated learning for end-edge platform

Anjir Ahmed Chowdhury[1,3], **S M Hasan Mahmud**[2,3], **Md Palash Uddin**[4,5], **Seifedine Kadry**[6*], **Jung-Yeon Kim**[7], **Yunyoung Nam**[7*]

1 Department of Computer Science, University of Houston, Houston, Texas, United States of America, **2** Department of Software Engineering, Daffodil International University, Daffodil Smart City (DSC), Birulia, Savar, Bangladesh, **3** Centre for Advanced Machine Learning and Applications (CAMLAs), Dhaka, Bangladesh, **4** Department of Computer Science and Engineering, Hajee Mohammad Danesh Science and Technology University, Dinajpur, Bangladesh, **5** School of Information Technology, Deakin University, Geelong, Victoria, Australia, **6** Department of Computer Science and Mathematics, Lebanese American University, Beirut, Lebanon, **7** Department of ICT Convergence, Soonchunhyang University, Asan, Korea

* skadry@gmail.com

OPEN ACCESS

Citation: Chowdhury AA, Hasan Mahmud SM, Palash Uddin Md, Kadry S, Kim J-Y, Nam Y (2025) Nuclei segmentation and classification from histopathology images using federated learning for end-edge platform. PLoS One 20(7): e0322749. https://doi.org/10.1371/journal.pone.0322749.

Editor: Jyotir Moy Chatterjee, Graphic Era Deemed to be University, INDIA

Received: February 17, 2024

Accepted: March 28, 2025

Published: July 10, 2025

Copyright: © 2025 Chowdhury et al. This is an open access article distributed under the terms of the Creative Commons Attribution License, which permits unrestricted use, distribution, and reproduction in any medium, provided the original author and source are credited.

Data availability statement: https://www.kaggle.com/datasets/theredlad/pannuke-dataset-experimental-data/data.

Funding: This work was supported by the National Research Foundation of Korea (NRF) grant funded by the Korean government (MSIT) (No. RS-2023-00218176 to YN) and the Soonchun-hyang University Research Fund (to YN).

Competing interests: The authors have declared that no competing interests exist.

Abstract

Accurate nuclei segmentation and classification in histology images are critical for cancer detection but remain challenging due to color inconsistency, blurry boundaries, and overlapping nuclei. Manual segmentation is time-consuming and labor-intensive, highlighting the need for efficient and scalable automated solutions. This study proposes a deep learning framework that combines segmentation and classification to enhance nuclei evaluation in histopathology images. The framework follows a two-stage approach: first, a SegNet model segments the nuclei regions, and then a DenseNet121 model classifies the segmented instances. Hyperparameter optimization using the Hyperband method enhances the performance of both models. To protect data privacy, the framework employs a FedAvg-based federated learning scheme, enabling decentralized training without exposing sensitive data. For efficient deployment on edge devices, full integer quantization is applied to reduce computational overhead while maintaining accuracy. Experimental results show that the SegNet model achieves 91.4% Mean Pixel Accuracy (MPA), 63% Mean Intersection over Union (MIoU), and 90.6% Frequency-Weighted IoU (FWIoU). The DenseNet121 classifier achieves 83% accuracy and a 67% Matthews Correlation Coefficient (MCC), surpassing state-of-the-art models. Post-quantization, both models exhibit performance gains of 1.3% and 1.0%, respectively. The proposed framework demonstrates high accuracy and efficiency, highlighting its potential for real-world clinical deployment in cancer diagnosis.

1 Introduction

Cancer is a malignant disorder characterized by abnormal growth and proliferation of nuclei, which can spread to other parts of the body, posing a significant threat to human health. Medical practitioners have used various imaging techniques for cancer screening for over

40 years, but biopsy remains the most accurate diagnostic method [1]. During the biopsy, tissue samples are stained to enhance their microscopic appearance, and histopathology images are analyzed to identify malignant regions through visual inspection. However, manual evaluation of stained histology slides is time-consuming, labor intensive, and subject to observer variability [2,3]. Consequently, the field of digital pathology (DP) is gaining attention by employing computer-assisted diagnosis (CAD) techniques to support pathologists and improve the efficiency of histopathology image analysis [4]. DP images, generated through tissue slicing, staining, and digitization, are typically high-resolution and may contain tens of thousands of nuclei with significant variations in color, texture, shape, and morphology [5]. Manual evaluation of such complex images is challenging, highlighting the need for automated segmentation, localization, and classification of different types of nuclei [6–9]. Nuclei segmentation is a crucial step in cancer diagnosis and prognosis, as it allows the extraction of interpretable features [10–14].

Over the past two decades, various nuclei segmentation methods have been proposed, which can be broadly categorized into a handcrafted feature (HF)-based and deep learning (DL)-based approaches. HF-based methods involve techniques such as filtering [16], thresholding [17], marker-controlled watershed, region accumulation [18], morphological operations [19], and graph cuts [19]. While these methods require manual feature design and tuning, they are often limited in effectiveness due to their reliance on predefined features. In contrast, DL-based methods automatically extract relevant features, making them more adaptable and effective. However, both HF and DL methods have strengths and limitations, and the choice depends on the specific dataset and task requirements (Fig 1).

Deep learning, particularly convolutional neural networks (CNNs), has shown significant promise in nuclei segmentation [20,21]. CNN-based methods can be classified into one-stage and two-stage approaches. Two-stage methods involve first detecting individual nuclei and then refining the segmentation. For example, Mask R-CNN [16] uses bounding boxes to locate nuclei instances but struggles with overlapping and occluded instances (Fig 1). SPA-Net [19] addresses this issue by detecting instance centroids and performing semantic segmentation in two stages. Similarly, BRP-Net [18] generates region proposals based on nuclei boundaries and refines the foreground mask. However, these two-stage methods often have high complexity and are not suited for end-to-end training. In contrast, one-stage methods like U-Net [22] use a single network to predict instance masks directly. Micro-Net [23], an improved version of U-Net, processes input at different resolutions, making it more robust for varying nuclei sizes. DCAN [20] generates separate maps for nuclei contours and clusters, improving boundary detection. BES-Net [24] and CIA-Net [25] further enhance information flow between decoder layers to refine segmentation quality.

The proposed framework improves nuclei identification through a two-stage segmentation-based classification approach. The first stage focuses on detecting nuclei regions using segmentation models such as Fully Convolutional Networks (FCN), U-Net, SegNet, and ResUnet. The second stage classifies the segmented instances using models like VGG16, VGG19, ResNet50, DenseNet121, and InceptionV2. Hyperparameter tuning using the Hyperband algorithm optimizes both segmentation and classification performance. To ensure data privacy, FedAvg is integrated, enabling collaborative training across devices without sharing raw data. For deployment on edge devices, post-training quantization techniques, including dynamic range, full integer, and float16 quantization, are applied to improve model efficiency and reduce computational overhead.

The key objectives of this study are:

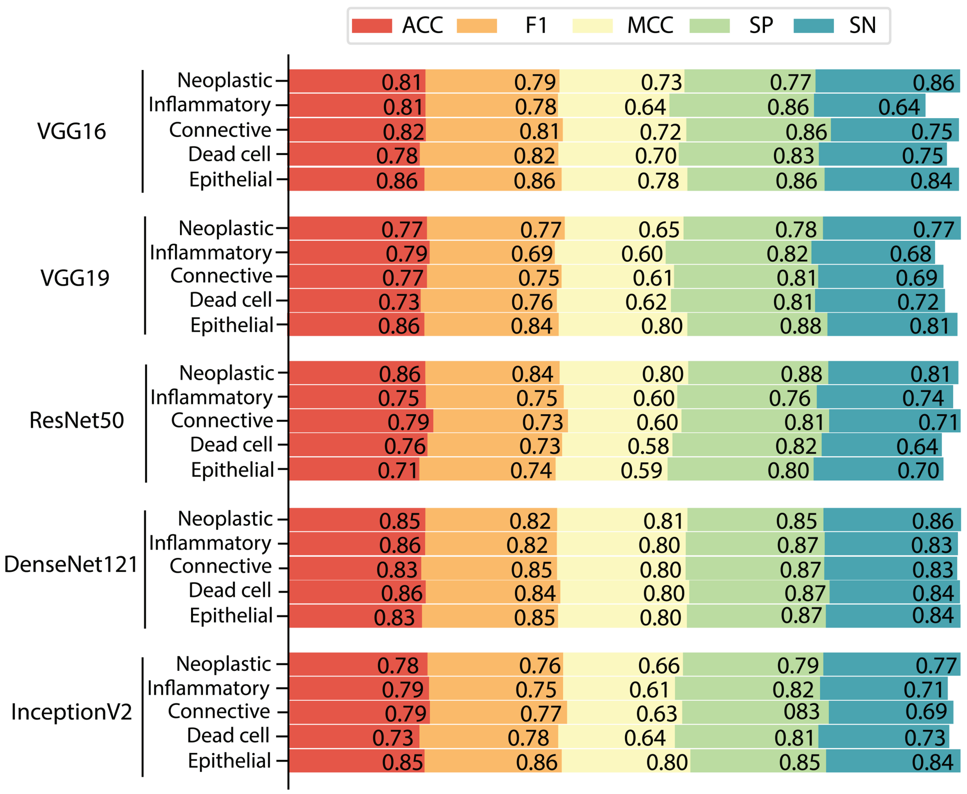

Fig 1. Pre-process PanNuke dataset [15] for nuclei instance segmentation and classification.

https://doi.org/10.1371/journal.pone.0322749.g001

- **Refined Nucleus Identification:** Develop a two-stage segmentation-based approach to accurately identify and segment nuclei in stained histopathology images.
- **Automated Segmentation and Classification:** Use segmentation models (FCN, U-Net, SegNet, ResUnet) and classification models (VGG16, VGG19, ResNet50, DenseNet121, InceptionV2) to automate nuclei analysis.
- **Performance Optimization:** Apply the Hyperband algorithm for hyperparameter tuning to improve the accuracy and efficiency of both segmentation and classification models.
- **Privacy-Preserving Training:** Incorporate federated learning to enable collaborative training without sharing sensitive patient data.
- **Edge Deployment:** Optimize the model using post-training quantization techniques to enable efficient real-time performance on resource-constrained edge devices.

The main contributions of this work are:

1. **Efficient Fully Automated Framework:** A fully automated deep learning framework for nuclei segmentation and classification that addresses challenges such as color inconsistency, blurry boundaries, and overlapping instances.
2. **Enhanced Performance Through Optimization and Privacy:** Hyperband-based tuning enhances performance, while FedAvg ensures data privacy by enabling decentralized training without exposing raw data.
3. **Edge Device Deployment:** The optimized models are customized for deployment on edge devices, ensuring real-time performance and efficient resource utilization.

The remainder of the paper is structured as follows: Sect 2 describes the network architecture and methods used. Sect 3 presents the experimental setup and results, including an analysis of model performance under different configurations. Sect 6 concludes the paper and outlines future research directions.

2 Methods & materials

2.1 Methodology overview

The proposed framework consists of two main stages for nuclei segmentation and classification. In the first stage, pre-processed datasets are fed into segmentation models to identify individual nuclei instances. In the second stage, the segmented instances are passed to a classification model to determine the type of nucleus. Both the segmentation and classification models are optimized using the Hyperband algorithm for improved performance. After optimization, the models are quantized for efficient deployment on edge devices. The quantized models are then integrated with a federated learning algorithm to enable privacy-preserving training without sharing raw data. Fig 2 illustrates an overview of the proposed framework.

2.2 Dataset

The PanNuke Dataset [26] is a comprehensive collection of over 20,000 annotated microscopy images, including both hematoxylin and eosin (H&E) stained slides and immunofluorescent

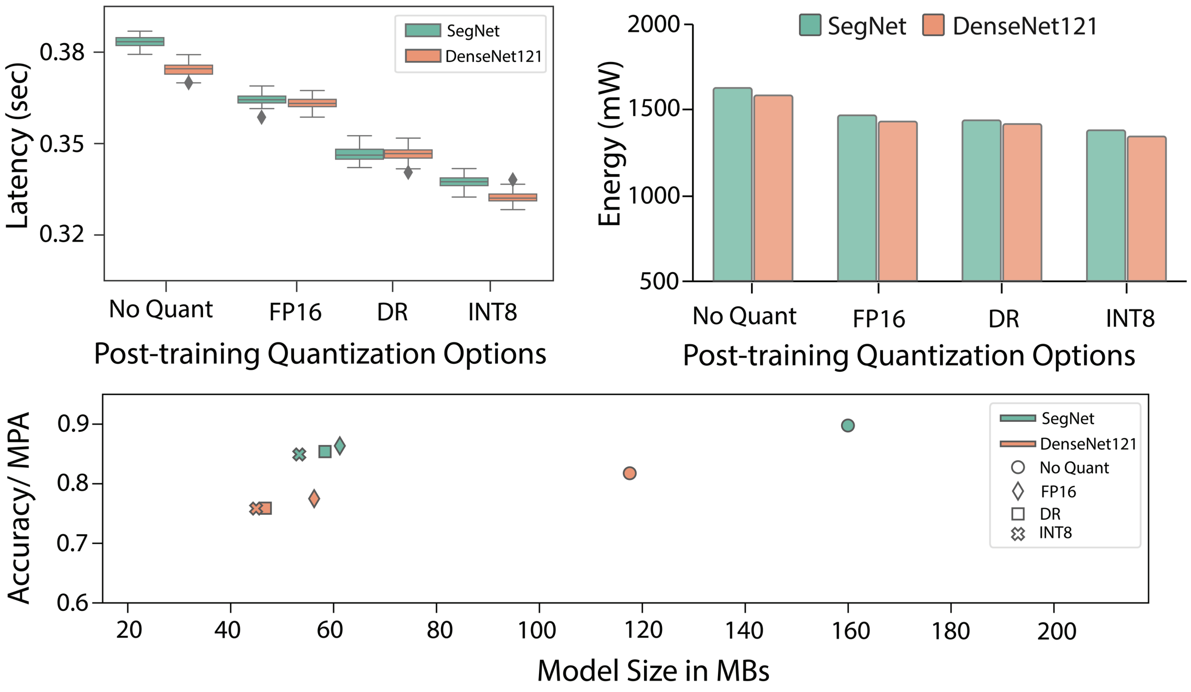

Fig 2. An Overview of the proposed framework. Different medical institutions locally train models on private data in two stages: segmentation followed by classification, with hyperparameter optimization applied to optimize the performance of both models. The optimized models are then quantized to reduce size and improve efficiency before being uploaded to a federated server. The server aggregates the quantized models, creating a robust, generalized model. Medical practitioners can download the aggregated model to improve healthcare insights while ensuring data privacy.

https://doi.org/10.1371/journal.pone.0322749.g002

images. Curated by researchers at the National Institutes of Health (NIH), this dataset encompasses a wide variety of tissue and nuclei types, divided into five main classes: neoplastic, non-neoplastic epithelial, connective, inflammatory, and dead nucleus. It serves as a valuable resource for developing and evaluating algorithms for nucleus segmentation and classification in diverse biological contexts. The dataset features high-quality annotations that allow for accurate performance assessments and comparisons with state-of-the-art methods. The distribution of nuclei varies across different tissue types (Fig 3). For instance, breast tissue contains the highest number of nuclei (51,077), followed by colon tissue (35,711), while bladder tissue has the fewest (2,839). Among the different classes, neoplastic tissue exhibits the highest total number of nuclei (77,403), followed by connective tissue (50,585), inflammatory tissue (32,276), and epithelial tissue (26,572). The dead nucleus class has the lowest count with only 2,908 nuclei.

2.3 Segmentation models

Fully Convolutional Networks (FCN) FCN [28] are a type of Convolutional Neural Network (CNN) designed for image segmentation tasks. FCNs replace the fully connected layers of traditional CNNs with convolutional layers, allowing them to produce dense per-pixel predictions, such as segmentation maps. The original FCN architecture used a VGG-16 network pretrained on ImageNet as the encoder, followed by an upsampling decoder to generate the final segmentation map. Subsequent improvements included the incorporation of dilated convolutions [29] and attention mechanisms [30] to enhance performance, particularly in medical image segmentation. A key feature of FCNs is the use of skip connections that combine low-level and high-level features through 1x1 convolutions, allowing for better spatial localization and improved segmentation accuracy.

U-Net U-Net [22] has become a popular model in medical image analysis, especially for segmentation tasks. The architecture consists of a contracting path, which reduces spatial dimensions through convolution and pooling layers, and an expanding path, which increases spatial resolution through upsampling. A defining characteristic of U-Net is its use of skip connections that link corresponding layers in the contracting and expanding paths, preserving fine-grained spatial information and enhancing segmentation accuracy. U-Net's success in

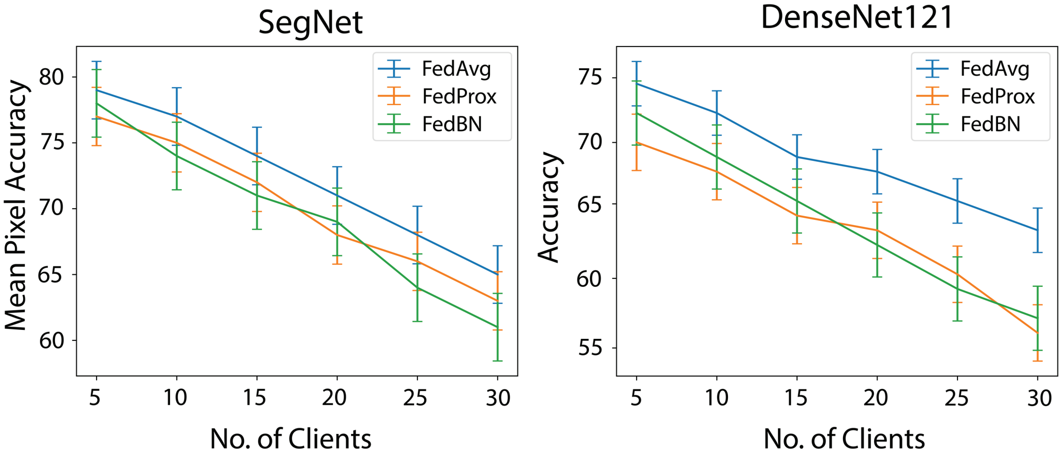

Fig 3. An overview of the PanNuke nuclei distribution across the nineteen tissue types, sorted by the total number of nuclei within each tissue. The total number of nuclei for each tissue type is provided in parentheses. Adapted from [27].

https://doi.org/10.1371/journal.pone.0322749.g003

segmenting complex structures, such as neuronal images, has led to its widespread adoption, including its notable performance in the 2018 Data Science Bowl [31].

SegNet SegNet [32] is another deep learning architecture designed for semantic image segmentation. It consists of an encoder-decoder structure similar to U-Net but introduces a unique "decoder index" mapping. This feature stores pooling indices from the encoder and uses them during decoding to guide the upsampling process, preserving fine-grained spatial details. SegNet has demonstrated efficient real-time processing in applications such as autonomous driving and medical image analysis, balancing accuracy with computational efficiency.

ResUnet ResUnet [33] is an extension of the U-Net architecture designed to improve segmentation performance by incorporating residual connections. These connections allow the network to learn residual functions, helping to mitigate the vanishing gradient problem and enabling more effective training. The addition of residual connections allows ResUnet to capture important details that might be lost in deeper layers. This makes ResUnet particularly suitable for complex image segmentation tasks, such as nucleus segmentation in biomedical images, where both fine-grained details and high-level semantic information are crucial.

2.4 Classification models

VGG The VGG network [34] is a widely used convolutional neural network (CNN) architecture for various image classification tasks, including nuclei classification in histopathology images. It has variants such as VGG-16 and VGG-19, distinguished by the number of layers—16 layers for VGG-16 and 19 layers for VGG-19. Both variants use small 3x3 convolutional filters to capture local spatial information while maintaining a manageable parameter count. VGG networks utilize 2x2 max-pooling layers with a stride of 2 to downsample feature maps and reduce spatial resolution. This structure enables VGG models to capture hierarchical features effectively, contributing to strong performance in image classification tasks, including nuclei classification.

ResNet50 ResNet50 [35] is a variant of the ResNet architecture, designed to address challenges in training very deep CNNs by introducing skip (residual) connections. Comprising 50 layers, ResNet50 incorporates convolutional layers with 3x3 and 1x1 filters, batch normalization layers, and fully connected layers. The skip connections enable the network to bypass certain layers, facilitating the learning of residual functions and alleviating the vanishing gradient problem. This architecture improves gradient flow and stabilizes training, making ResNet50 effective for image classification tasks, such as nuclei classification in histopathology images.

DenseNet121 DenseNet121 [36] is a variant of the DenseNet architecture known for its dense connectivity between layers, where each layer receives input from all preceding layers. This design addresses the vanishing gradient problem and promotes feature reuse, allowing for deeper and more trainable networks. DenseNet121 is composed of 121 layers, including convolutional and transition layers. The convolutional layers use 3x3 filters to extract features, while transition layers use 1x1 convolutions and 2x2 average pooling for downsampling. The dense connections ensure efficient gradient flow and information propagation, making DenseNet121 a powerful choice for image classification tasks, such as nuclei classification in histopathology.

InceptionV2 InceptionV2 [37] enhances the original Inception architecture with multi-scale processing, allowing the network to capture features at various scales. This is achieved through the use of convolutional filters of different sizes and pooling operations of varying dimensions. InceptionV2 also incorporates 1x1 convolutional filters, which combine outputs

from multiple filters, enhancing feature learning. These filters are part of the inception module, a key building block of the architecture. InceptionV2's ability to learn multi-scale features makes it effective for tasks like nuclei detection and classification in histopathological images.

2.5 Hyperparameter optimization with hyperband

Li et al. [38] introduced the Hyperband algorithm to accelerate the Random Search method for hyperparameter optimization. Hyperband achieves this by employing adaptive resource allocation and early-stopping techniques. It reformulates the hyperparameter optimization problem as a non-stochastic, exploratory infinite-arm bandit problem. Instead of training all configurations until the final epoch, Hyperband efficiently allocates resources to randomly selected hyperparameter configurations and discards unpromising ones early on.

Hyperband is an extension of the Successive Halving algorithm [39], which addresses best-arm identification in multi-armed bandit problems. In Successive Halving, the hyperparameter optimization problem is treated as a non-stochastic best-arm identification problem, where each arm represents a specific hyperparameter setting. The algorithm begins by allocating a budget, denoted as B, uniformly across n configurations. After a fixed number of training iterations, the performance of each configuration is evaluated using an intermediate loss on a holdout set. The worst-performing half of the configurations is discarded, and the process is repeated until only one configuration remains.

To use the Hyperband algorithm, two input values are required: R and η. R represents the maximum resource that can be allocated to a configuration, and η indicates the ratio of configurations eliminated in each iteration of Successive Halving. These values combine to guide the resource allocation and early-stopping decisions in Hyperband.

$$S_{max} = log_n(R) \tag{1}$$

2.6 Optimizing model for cloud and edge deployment through quantization

Quantization is a widely used technique in deep learning that reduces the memory and computational requirements of neural networks by lowering the precision of weights and activations from the standard 32-bit floating-point representation to lower-bit-width formats. While this reduction in precision helps alleviate resource constraints, it may lead to accuracy degradation due to the information loss during quantization. To mitigate this issue, post-quantization methods such as post-quantization training have been proposed. In post-quantization training, the quantized model is fine-tuned on a dataset, where the weights and activations are updated to minimize the loss function, improving the model's accuracy without sacrificing the benefits of reduced computational and memory requirements.

This is especially important for deploying deep learning models on edge-computing platforms, which face constraints like limited memory, on-chip resources, and battery capacity. To overcome these limitations, the network architecture must be lightweight, ensuring acceptable accuracy and speed while consuming minimal power.

Two primary approaches for quantizing neural networks are Post-Training Quantization (PTQ) and Quantization-Aware Training (QAT) [40]. PTQ fine-tunes a pre-trained network with reduced precision, whereas QAT incorporates the quantization process during training, enabling the network to learn optimal quantized representations. Both methods are effective in reducing the memory and computational requirements of neural networks while maintaining accuracy, making them essential for deploying models on resource-constrained edge devices.

2.7 Federated learning

Federated learning (FL) [41] has emerged as a promising approach for preserving privacy when applying machine learning to medical data. It has been successfully applied to various healthcare tasks, including predicting patient outcomes [42], medication adherence [43], hospital readmission [44], disease risk [45], and detecting chronic diseases such as diabetes [46]. FL has also enabled the creation of large-scale annotated medical datasets [47] and has been proposed as a solution for safeguarding data privacy in genomics research [48].

In FL, the model is trained on decentralized datasets, meaning that raw data is never transmitted to a central location. Instead, training occurs locally on each participating device or server, and the locally trained models are then aggregated to form a global model. This approach ensures that sensitive medical data remains private and confidential, a critical concern in healthcare.

While ensemble learning often uses centralized data and model subsets, raising privacy concerns, FL offers a more secure alternative. By utilizing distributed data and device-trained models, FL reduces the risk of privacy breaches and communication overhead. Given the sensitive nature of medical data and the need for collaboration across institutions, we opted for FL rather than centralized training with ensemble learning.

In typical FL setups, stochastic gradient descent (SGD) is used to train the model. Each device computes the gradients of the local loss function concerning the model parameters, and the global model is updated by averaging these gradients across all devices. This decentralized process ensures that privacy is maintained throughout the training.

$$\theta_{t+1} = \theta_t - \eta \frac{1}{n} \sum_{i=1}^{n} \nabla f_i(\theta_t) \tag{2}$$

where θ_t is the global model parameters at iteration t, η is the learning rate, and f_i is the local loss function for the ith participating device.

2.8 Implementation details

The nuclei segmentation and classification models were developed using TensorFlow 2.2, with CUDA 10.1 and cuDNN 7.5.0 for GPU acceleration. The training process employed SGD to achieve faster convergence. For the binary classification task, the segmentation network was designed to classify individual pixels in the image. It utilized mean squared error (MSE) loss for bounding box regression and a cross-entropy loss function for single-pixel binary classification. The segmentation task involved pixel classification for precise segmentation while bounding boxes were used for object localization, a common approach in instance segmentation. Bounding boxes help to identify individual nuclei, and pixel classification refines object boundaries.

The two networks were trained sequentially: the segmentation network was trained first, and then the decision network was trained with the segmentation network's weights frozen. The decision network only fine-tuned its weights during training, applying transfer learning to mitigate the risk of overfitting.

Both networks were trained using SGD with a learning rate of 0.001 and a cross-entropy loss rate of 0.1. Due to GPU memory constraints and the large image size, the batch size was set to 2. The PanNuke dataset was split into 80% for training and 20% for testing, with 5-fold cross-validation. To optimize both models, we used the Hyperband algorithm implemented with Keras Tuner [49], as detailed in Table 1. Additionally, federated learning (FL) algorithms were implemented using TensorFlow Federated [50].

Table 1. Search space for segmentation and classification models.

Search Space	Range	Search Space	Range
**Segmentation Model**		**Classification Model**	
No. of Filters	[32,64,128,256]	No. of Units	[16, 32,64,128,256]
Filter Length	[10,15,20,25,30]	Learning Rate	[0.0001, 0.001, 0.01]
Filter Width	[2,4,8,16]	Optimizer	['Rmsprop', 'Adam', 'SGD']
Initialization Mode	['uniform', 'Normal', 'zero', 'he_uniform']	Regularizer	['L1', 'L2']
Activation Function	['softmax', 'relu', 'sigmoid', 'tanh']	Output Activation function	['softmax', 'relu', 'tanh']
Neurons in Fully Connected Layer	[16,32,64,128]	Dense Units	[64,128,256]
Batch Size	[32,64,128]	Batch Size	[32,64,128]
dropout	[0.05,0.1,0.15,0.2,0.25]	dropout	[0,0.2,0.3]

https://doi.org/10.1371/journal.pone.0322749.t001

All experiments were conducted on an Ubuntu 22.04.2 LTS system with an Intel Core i7-14700KF CPU, 32GB of memory, and an Nvidia RTX 3070 GPU. For deployment on an edge device, we used the NVIDIA Jetson Nano [51], featuring a quad-core A57 processor, 2GB of LPDDR4 memory, and a 128-core NVIDIA Maxwell GPU. Before deployment, the model was quantized using the TensorFlow Lite converter to optimize it for the edge device.

2.9 Evaluation metrics

2.9.1 Segmentation. Nucleus extraction, a type of semantic segmentation, involves pixel-level classification, where each pixel is assigned to a specific class or category. The performance of semantic segmentation models is often quantitatively evaluated using several metrics, including Mean Pixel Accuracy (MPA), Mean Intersection over Union (MIoU), and Frequency Weighted Intersection over Union (FWIoU). For nucleus segmentation specifically, additional metrics like Class Pixel Accuracy (CPA) and Intersection over Union (IoU) are commonly used to provide a more detailed evaluation.

Pixel Accuracy is one of the most widely used metrics in semantic segmentation. It measures the accuracy at the pixel level for each class in the segmented image. By computing the accuracy for each class separately, this metric provides a more nuanced understanding of how well the model performs for specific categories. This allows for a better assessment of which classes the model excels at segmenting and which classes it struggles with. Analyzing accuracy on a per-class basis is invaluable for identifying areas of improvement in the model's training process, data augmentation strategies, or architecture. It provides targeted insights into the model's strengths and weaknesses, which can guide efforts to enhance performance for challenging classes.

The following formulas are used to calculate these various evaluation metrics:

$$CPA = \frac{\sum_{i=0}^{n} P_{ii}}{\sum_{i=0}^{n} \sum_{j=0}^{n} P_{ij}} \tag{3}$$

$$MPA = \frac{1}{n+1} \sum_{i=0}^{n} \frac{P_{ij}}{\sum_{j=0}^{n} P_{ij}} \tag{4}$$

$$IoU = \frac{\sum_{j=0}^{n} P_{ii}}{\sum_{i=0}^{n} \sum_{j=0}^{n} P_{ij} + \sum_{i=0}^{n} \sum_{j=0}^{n} P_{ji} - P_{ii}} \tag{5}$$

$$MIoU = \frac{1}{n+1}\sum_{i=0}^{n}\frac{P_{ii}}{\sum_{j=0}^{n}P_{ij}+\sum_{j=0}^{n}P_{ji}-P_{ii}} \quad (6)$$

$$FWIoU = \frac{1}{\sum_{i=0}^{n}\sum_{j=0}^{n}P_{ij}}\sum_{i=0}^{n}\frac{P_{ii}}{\sum_{j=0}^{n}P_{ij}+\sum_{j=0}^{n}P_{ji}-P_{ii}} \quad (7)$$

To ensure a fair evaluation of instance segmentation performance, we utilize the Panoptic Quality (PQ) as the assessment metric, as recommended in previous studies [15,52]. PQ is a commonly used evaluation metric for panoptic segmentation tasks. It was initially introduced for nuclei segmentation, and it is defined as follows:

$$PQ = \frac{|TP|}{|TP|+\frac{1}{2}|FP|+\frac{1}{2}|FN|}\times\frac{\sum_{(x,y)\in TP}IoU(x,y)}{|TP|} \quad (8)$$

In addition, we provide a breakdown of the Panoptic Quality (PQ) performance for all 19 tissues in terms of multi-class PQs (mPQs) and binary PQs (bPQs). The mPQs represent the average PQ score for each of the five nucleus categories, while the bPQs calculate the overall performance on images containing all five categories. We selected an IoU threshold of 0.5 to determine true positives during the PQ calculation.

2.9.2 Classification. Five performance metrics are commonly used to evaluate the classification of different nuclei: Sensitivity (SN), Specificity (SP), Accuracy (ACC), F1-Score, and Matthews Correlation Coefficient (MCC). These metrics are formulated as follows:

$$SN = \frac{TP}{TP+FN} \quad (9)$$

$$SP = \frac{TN}{FP+TN} \quad (10)$$

$$ACC = \frac{TP+TN}{TP+FN+FP+TN} \quad (11)$$

$$F-Score = \frac{2TP}{2TP+FP+FN} \quad (12)$$

$$MCC = \frac{(TP\times TN)-(FP\times FN)}{\sqrt{(TP+FP)(TP+FN)(TN+FP)(TN+FN)}} \quad (13)$$

3 Result and analysis

3.1 Segmentation model evaluation

The experimental results are based on the 20% testing set. As shown in Table 2, SegNet achieved the highest scores in MPA (91.4%), MIoU (63.5%), and FWIoU (90.8%). ResUnet followed closely, with MPA and MIoU scores of 91.2% and 62.6%, respectively. While the performance differences between the models are minimal, other factors such as CPA and IoU should be considered when selecting the optimal model for nucleus segmentation.

The manual segmentation approach struggles with issues like color inconsistency, blurry nuclei, and overlapping nuclei, which are common in several images from the Panuke dataset. In contrast, SegNet, a CNN-based segmentation model, effectively addresses these challenges,

Table 2. Quantitative evaluation of different segmentation models.

Models	MPA (%)	MIoU (%)	FWIoU (%)
FCN	90.2	61.2	90.6
U-Net	89.8	59.9	88.7
SegNet	91.4	63.5	90.8
ResUnet	91.2	62.6	90.2

https://doi.org/10.1371/journal.pone.0322749.t002

Table 3. CPA and IoU comparison of different segmentation models.

Models	CPA (%)	IoU (%)
FCN	37.6	25.4
U-Net	32.8	29.7
SegNet	44.7	35.5
ResUnet	40.6	32.8

https://doi.org/10.1371/journal.pone.0322749.t003

achieving impressive performance metrics: 91.4% MPA, 63.5% MIoU, and 90.8% FWIoU. These results highlight SegNet's strong capability in handling typical segmentation issues, making it a solid choice for nucleus segmentation tasks.

The evaluation metrics used to compare the models in Table 3 are Class Pixel Accuracy (CPA) and Intersection over Union (IoU). The table indicates that SegNet outperforms the other models, achieving an average pixel accuracy of 44.7 and an IoU of 35.5. U-Net also demonstrates strong performance, with an average pixel accuracy of 32.8 and an IoU of 29.7. FCN and ResUnet exhibit similar performance, with average pixel accuracies of 37.6 and 40.6, respectively. However, ResUnet has a lower IoU score of 32.8, compared to FCN's score of 25.4.

For each of the 19 tissues, we calculated both multi-class (mPQ) and binary (bPQ) panoptic qualities, which were adopted from [52]. The results of our experiment, as presented in Table 4, demonstrate that SegNet consistently outperforms ResUnet for both mPQ and bPQ evaluation metrics. SegNet achieves better overall and tissue-specific performance for the mPQ metric than any other state-of-the-art model.

Table 5 presents the average PQ for each type of nucleus in the PanNuke dataset. SegNet outperforms all other state-of-the-art models in the neoplastic, connective, and epithelial nuclei categories. However, ResUnet excels in the inflammatory and dead nuclei categories. Notably, dead nuclei consistently achieve the lowest PQ across all models. This could be attributed to the class imbalance in the dataset, as the small number and size of dead nuclei make it difficult to achieve an IoU greater than 0.5 for true positives, leading to poorer performance.

3.2 Classification model evaluation

In Table 6, DenseNet121 achieved the highest values across all evaluation metrics, including Sensitivity (0.84), Specificity (0.86), F1-Score (0.83), and MCC (0.67). It also had the highest accuracy at 0.83. VGG19 closely followed, with Sensitivity of 0.81, Specificity of 0.83, and an F1-Score of 0.81. InceptionV2 also performed well, achieving a Sensitivity of 0.82, Specificity of 0.85, and an F1-Score of 0.81. VGG16 had an F1-Score of 0.80, while ResNet50 recorded the lowest values for all metrics: Sensitivity (0.79), Specificity (0.80), F1-Score (0.79), MCC (0.61), and accuracy (0.75).

Table 4. Average mPQ and bPQ of the nuclei labels across the 19 different tissue types in the PanNuke dataset.

Tissue	FCN		U-Net		SegNet		ResUNet	
	mPQ	bPQ	mPQ	bPQ	mPQ	bPQ	mPQ	bPQ
Adrenal	0.273	0.468	0.287	0.451	0.303	0.498	0.289	0.478
Bile Duct	0.312	0.457	0.321	0.425	0.333	0.512	0.312	0.492
Bladder	0.322	0.431	0.314	0.471	0.349	0.501	0.304	0.487
Breast	0.286	0.426	0.289	0.465	0.311	0.471	0.297	0.468
Cervix	0.333	0.487	0.324	0.493	0.325	0.508	0.314	0.486
Colon	0.312	0.486	0.301	0.432	0.319	0.472	0.298	0.461
Esophagus	0.298	0.451	0.311	0.441	0.306	0.492	0.279	0.483
H&N	0.354	0.487	0.334	0.472	0.359	0.511	0.311	0.492
Kidney	0.322	0.462	0.317	0.456	0.327	0.489	0.321	0.463
Liver	0.312	0.432	0.322	0.441	0.331	0.478	0.300	0.453
Lung	0.307	0.478	0.298	0.422	0.317	0.511	0.305	0.486
Ovarian	0.293	0.464	0.299	0.431	0.308	0.498	0.299	0.468
Pancreatic	0.302	0.457	0.312	0.446	0.337	0.510	0.311	0.502
Prostate	0.311	0.495	0.297	0.472	0.322	0.521	0.313	0.502
Skin	0.276	0.431	0.299	0.421	0.314	0.467	0.307	0.431
Stomach	0.297	0.421	0.301	0.431	0.327	0.479	0.311	0.453
Testis	0.267	0.412	0.287	0.403	0.297	0.467	0.290	0.449
Thyroid	0.302	0.436	0.299	0.441	0.312	0.498	0.316	0.477
Uterus	0.312	0.425	0.311	0.431	0.338	0.511	0.324	0.501

https://doi.org/10.1371/journal.pone.0322749.t004

Table 5. Average PQ of each nucleus category.

Models	Neoplastic	Inflammatory	Connective	Dead nuclei	Epithelial
FCN	0.412	0.431	0.337	0.121	0.471
UNet	0.451	0.446	0.434	0.231	0.421
SegNet	0.512	0.487	0.511	0.333	0.441
ResUnet	0.498	0.501	0.486	0.356	0.432

https://doi.org/10.1371/journal.pone.0322749.t005

Table 6. Specificity (SP), sensitivity (SN), F1-Score, Matthews correlation coefficient (MCC), and accuracy (ACC) for classification of nuclei types.

Models	SP	SN	F1	MCC	ACC
VGG16	0.79	0.81	0.80	0.63	0.77
VGG19	0.81	0.83	0.81	0.66	0.79
ResNet50	0.79	0.80	0.79	0.61	0.75
DenseNet121	0.84	0.86	0.83	0.67	0.83
InceptionV2	0.82	0.85	0.82	0.66	0.81

https://doi.org/10.1371/journal.pone.0322749.t006

DenseNet121 outperformed the other models in all evaluation metrics, making it the most effective model for the task at hand. VGG19 and InceptionV2 also performed well, achieving high scores across most metrics. In contrast, ResNet50 consistently had the lowest performance across all evaluated criteria. Fig 4 provides a visual representation of the class-wise performance of the classification models.

3.3 Hyperparameter optimization

The Hyperband algorithm is employed for hyperparameter optimization to identify the optimal architecture for both the segmentation and classification models. This is implemented using Keras Tuner [49], which efficiently searches for the best hyperparameter configurations. The search space for both Segmentation and Classification models is provided in Table 1. The

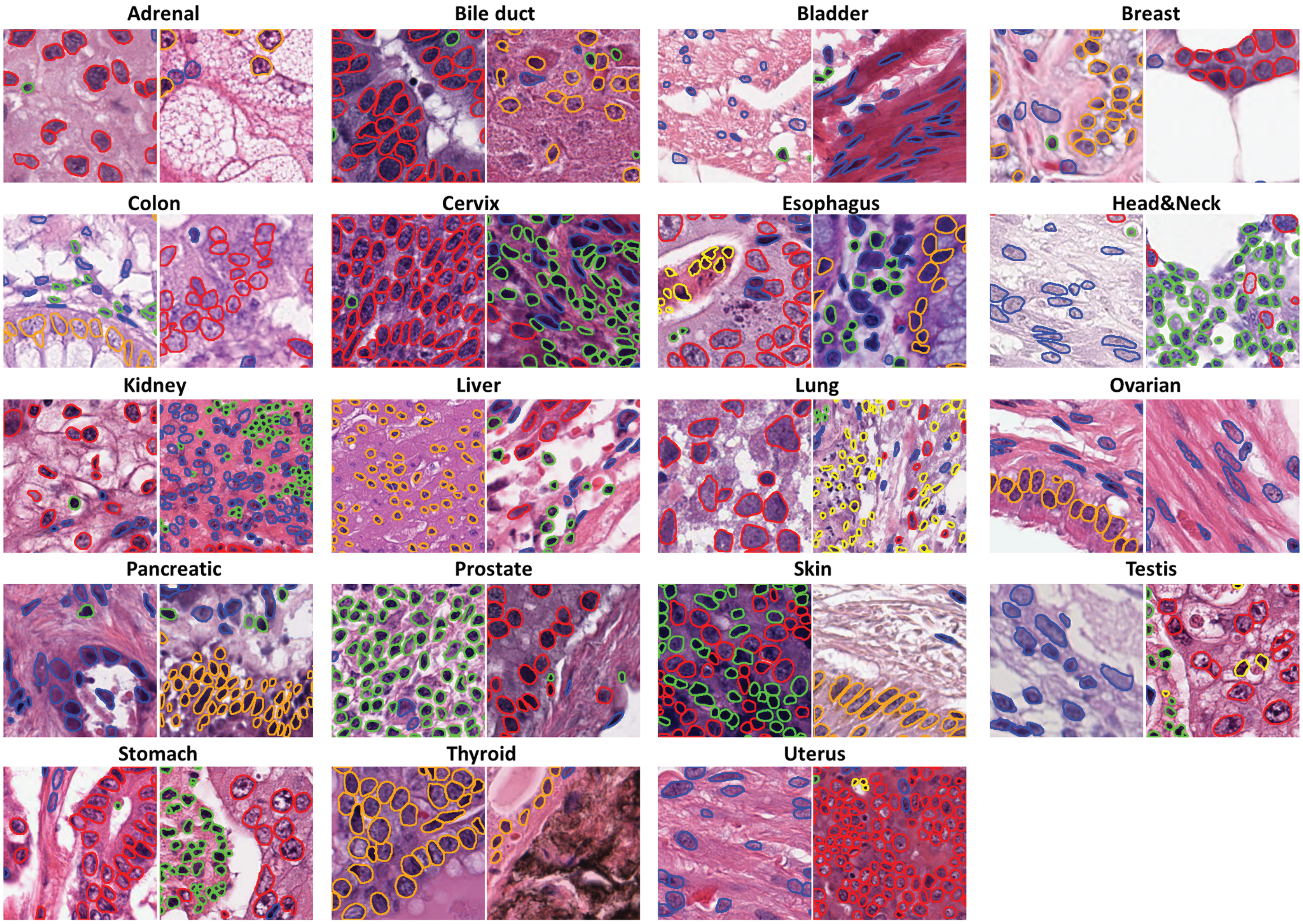

Fig 4. Class-wise performance of the classification models for categorizing nucleus.

https://doi.org/10.1371/journal.pone.0322749.g004

performance of various architectures is illustrated in Fig 5. While many architectures performed similarly, some did not converge during the search process. The best combination of hyperparameters was selected based on Mean Pixel Accuracy (MPA) for the SegNet model and Accuracy for the DenseNet121 model. Both models showed an improvement of 2% with the selected hyperparameters.

3.4 Quantization

Following the optimization process, we performed quantization of the SegNet and DenseNet121 models using FP16, dynamic range, and INT8 techniques. We then evaluated the models' inference latency, model size, accuracy, and energy consumption, as shown in Fig 6. The results reveal that both models experienced reduced latency, smaller model sizes, and lower energy consumption when quantized using the INT8 technique, though at the cost of a slight reduction in accuracy. Notably, INT8 quantization resulted in the smallest model size.

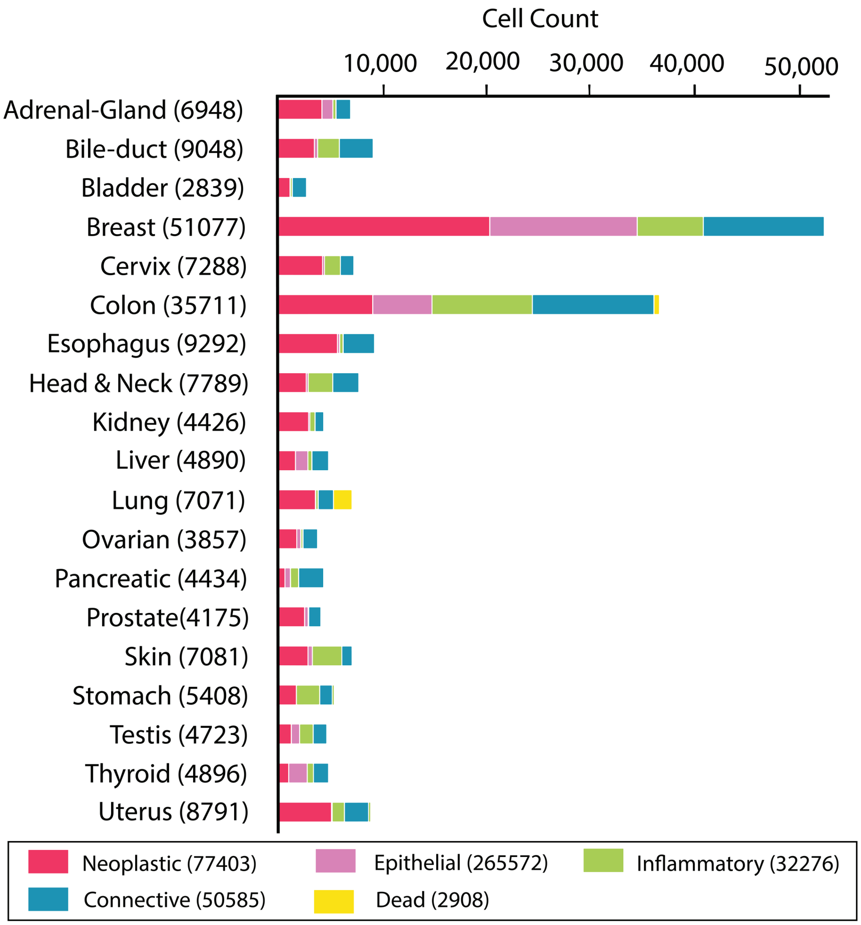

Fig 5. Performance of the different combinations for the SegNet and the DenseNet121 models.

https://doi.org/10.1371/journal.pone.0322749.g005

To further assess the impact of quantization, we deployed the models on the Nvidia Jetson Nano, utilizing the MAXN (10 Watt) power mode on the Jetson Nano board. No external I/O or peripherals were connected during the measurements. Since the Jetson Nano (2 GB version) does not have an INA3221 power monitoring interface, we measured energy consumption using a digital multimeter. As shown in Fig 6, INT8 quantization led to lower energy consumption with only a minor decrease in accuracy. This reduction in energy consumption is particularly significant, as high energy usage is a key limitation for edge devices.

3.5 Federated learning

To compare the performance of two models that underwent quantization using INT8 techniques, a distributed learning approach was employed. Various state-of-the-art Federated Learning (FL) algorithms, including FedAvg, FedProx, and FedBN, were selected for comparison against centralized learning. The results of these experiments are presented in Table 7.

From the table, it is evident that FedAvg outperformed the other FL algorithms in both models. Specifically, FedAvg achieved a 4% increase in accuracy compared to FedProx and a 2% increase compared to FedBN. Additionally, FedAvg demonstrated a 1% and 2% increase

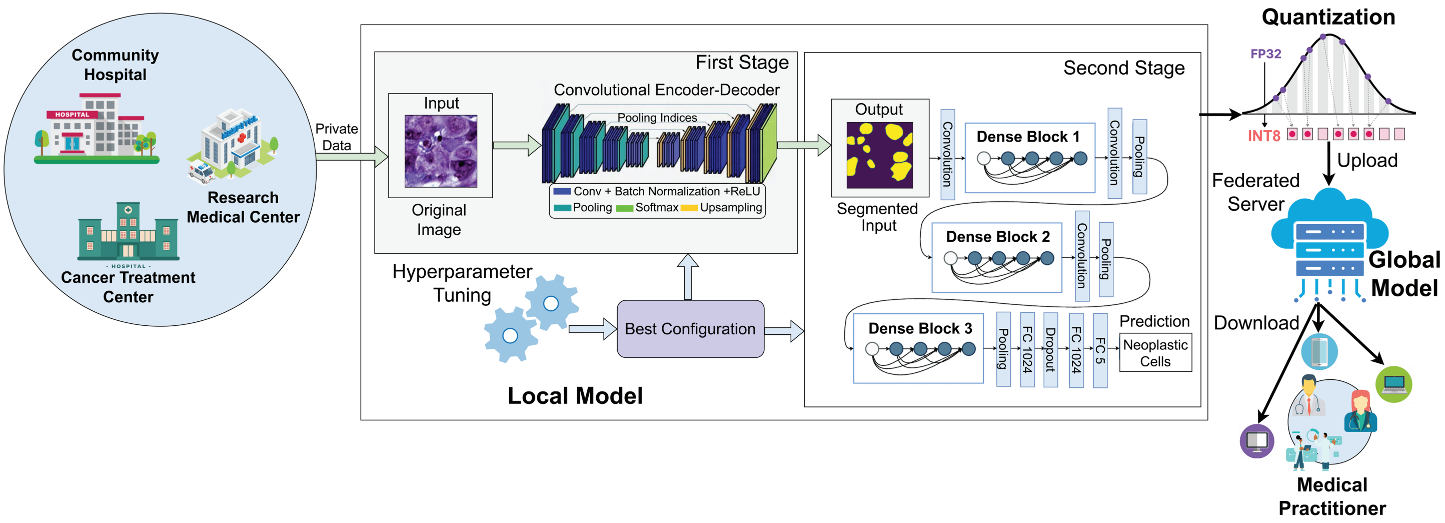

Fig 6. Performance analysis of different quantization techniques on latency, energy consumption, and size.

https://doi.org/10.1371/journal.pone.0322749.g006

Table 7. Performance comparison of federated learning algorithms for nucleus segmentation with INT8 quantized models.

FL Methods	SegNet		DenseNet121	
	MPA	CPA	ACC	F1
Centralize Learning	91.4	44.7	0.83	0.83
Centralize Learning (Optimized)	92.7	44.9	0.84	0.83
Centralize Learning (Quantized)	83.7	38.2	0.78	0.77
FedAvg	79.9	36.3	0.71	0.70
FedProx	77.8	33.2	0.67	0.68
FedBN	78.5	34.6	0.69	0.69

https://doi.org/10.1371/journal.pone.0322749.t007

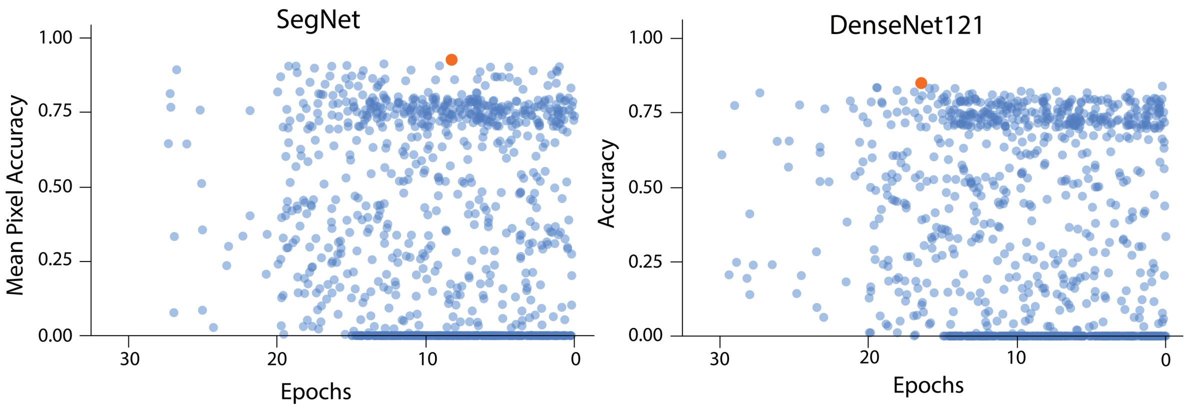

Fig 7. Performance analysis of different FL algorithms on both models.

https://doi.org/10.1371/journal.pone.0322749.g007

in Mean Pixel Accuracy (MPA) over FedBN and FedProx, respectively. These results suggest that FedAvg may be the most effective FL algorithm for nucleus segmentation in the given models.

Table 8. Compare the performance of the proposed framework with other state-of-the-art frameworks.

Reference	Year	Segmentation Model	Classification Model	Metrics
[53]	2020	W-Net	SC-CNN	a-PQ : 65%
[54]	2018	Trapezoidal LSTM	Trapezoidal LSTM	Precision : 96.64%, Recall : 96.79%, F1 : 96.71%
[55]	2022	Mobile-Net-v2 + squeeze-excitation sub-network	Feature distillation backbone	mPQ: 50%, bPQ: 63.7%
[56]	2019	Deep Residual Aggregation Network	ResNet50	Dice Score : 78%, ACC : 81%
Ours	2024	SegNet	DenseNet121	MPA : 91.4%, MIoU : 63.5%, FWIoU: 90.8%, ACC : 83%

https://doi.org/10.1371/journal.pone.0322749.t008

We also analyzed the impact of the number of clients during decentralized training. A larger number of clients can introduce conflicts among local gradients, which presents a significant challenge to the practicality of FL. To further investigate the efficacy of FedAvg compared to other FL algorithms in scenarios with varying numbers of clients, we simulated training with six smaller dataset partitions and five different client configurations: 10, 15, 20, 25, and 30 clients. The results, shown in Fig 7, reveal a consistent decline in testing accuracy as the number of clients increases. However, FedAvg exhibited a slower decline in accuracy compared to the other FL algorithms, demonstrating the robustness and scalability of FedAvg in scenarios with a higher number of clients.

3.6 Discussion and comparison with state-of-the-art frameworks

Table 8 summarizes the performance metrics of the proposed framework alongside other state-of-the-art models, providing insights into the strengths and weaknesses of each approach. We compare the performance of different segmentation models to evaluate their effectiveness.

Among the compared models, the W-Net model from [53] achieved an average pixel-wise precision of 65%, which, while reasonable, falls short compared to our proposed SegNet, which achieved an impressive Mean Pixel Accuracy (MPA) of 91.4%. This substantial improvement in MPA underscores the effectiveness of SegNet in accurately classifying pixels, making it a strong contender for segmentation tasks. Additionally, the Trapezoidal LSTM model introduced by [54] exhibited good performance, while Mobile-Net-v2 with a squeeze-excitation sub-network, proposed by [55], achieved an mPQ of 50% and bPQ of 63.7%. However, the proposed approach offers a lightweight solution that balances model complexity with performance, making it an appealing choice for practical applications.

In comparison, ResNet50, utilized by [56], achieved 81% accuracy, while DenseNet121 achieved 83% accuracy. Although these models performed well, they still lag behind SegNet in terms of segmentation performance.

It is important to note that the results presented in Table 8 are based on a specific dataset and experimental setup, meaning the relative performance of these models may vary depending on the dataset's characteristics, model hyperparameters, and other experimental conditions.

We intentionally did not incorporate both the fuzzy ensemble mechanism and transfer learning (TL) into our framework for several reasons. Our primary goal was to maintain the simplicity of our framework to ensure it could be deployed effectively on various edge platforms. By not combining these two approaches, we kept the model less complex, which aligns

with our deployment objectives. Furthermore, we recognized that fuzzy ensemble mechanisms and TL would require distinct data preprocessing and transformation techniques. Ensuring compatibility between fuzzy logic and TL could introduce challenges and may not result in optimal outcomes. Additionally, integrating fuzzy logic into TL would introduce extra hyperparameters that need careful tuning, increasing the risk of overfitting.

Several recent publications have evaluated models using the PanNuke dataset, including HoVer-UNet for nuclei instance segmentation [57] and CellViT, which employs Vision Transformers for automated instance segmentation [27]. However, in this work, we focus specifically on comparing models that utilize two-stage methods (segmentation followed by classification). Both of these recent studies represent significant advancements in leveraging transformer-based architectures for nuclei segmentation: HoVer-UNet offers a compact and efficient design, whereas CellViT explores the potential of large-scale, pre-trained Vision Transformers to achieve improved performance.

Given the two-stage nature of these models, evaluating their performance using a single metric becomes challenging, as each stage emphasizes different aspects of model performance. To maintain focus on the most relevant results, we selected only the best-performing CNN architectures for inclusion in this paper. Our comparison highlights the impact of multi-stage models on segmentation performance, considering key factors such as accuracy, inference time, and computational efficiency.

4 Ablation studies

We conducted ablation experiments to optimize the SegNet and DenseNet models for segmentation and classification tasks, respectively. These experiments involved exploring a predefined search space, as shown in Table 1. For the SegNet architecture, we varied several parameters, including the number of filters (32, 64, 128, 256), filter lengths (10, 15, 20, 25, 30), filter widths (2, 4, 8, 16), initialization modes ('uniform', 'normal', 'zero', 'he_uniform'), activation functions ('softmax', 'relu', 'sigmoid', 'tanh'), neurons in fully connected layers (16, 32, 64, 128), batch sizes (32, 64, 128), and dropout rates (0.05, 0.1, 0.15, 0.2, 0.25). Similarly, for the DenseNet model, which was used for classification, we adjusted the number of units (16, 32, 64, 128, 256), learning rates (0.0001, 0.001, 0.01), optimizers ('Rmsprop', 'Adam', 'SGD'), regularizers ('L1', 'L2'), output activation functions ('softmax', 'relu', 'tanh'), dense units (64, 128, 256), batch sizes (32, 64, 128), and dropout rates (0, 0.2, 0.3).

For both models, we evaluated key performance metrics such as accuracy, loss, and computational efficiency. Through iterative adjustments within the defined search space, we identified optimal configurations that enhanced model performance. Some configurations led to higher accuracy and lower loss, particularly those that utilized suitable activation functions, regularization techniques, and dropout rates. These ablation studies provided valuable insights into the sensitivity of SegNet and DenseNet architectures to various hyperparameters, offering a systematic approach to optimizing performance for segmentation and classification tasks.

5 Challenges, future directions and clinical impact

While our framework demonstrates promising results, several limitations should be acknowledged. A primary concern is the vulnerability of federated learning systems to attacks from malicious clients, potentially compromising the integrity of the shared model. Future research should focus on investigating effective mitigation strategies, such as robust aggregation methods, secure multi-party computation, and anomaly detection techniques, to enhance the robustness and reliability of federated learning models.

Another limitation is the absence of validation with domain experts in real-world clinical scenarios. Although our framework was thoroughly tested in simulated environments, real-world applications present challenges and complexities that simulations may not fully capture. Collaboration with domain experts for further evaluation in clinical settings will be crucial to ensuring the reliability and applicability of our framework in real-world medical practices.

The clinical benefits of automated nuclei segmentation using CNNs are substantial, offering the potential to significantly enhance disease diagnosis, treatment planning, and medical research. By delivering more accurate diagnostic tools and deeper insights into tissue morphology, our framework can play a pivotal role in improving healthcare outcomes. Moreover, automating image analysis through CNN-based segmentation increases diagnostic efficiency, facilitating quicker and more precise clinical decisions. This approach not only supports clinicians in making informed medical judgments but also contributes to scalable and effective healthcare solutions, ultimately transforming clinical practice in histopathology by enabling faster and more accurate diagnoses.

6 Conclusion

Nuclei segmentation plays a crucial role in tissue sample analysis, helping to identify and locate individual nuclei. This technique is especially important in the diagnosis of diseases like cancer and in the development of new treatments. In this study, we proposed a novel framework for nuclei segmentation and classification from pathology images. The framework leverages federated learning and quantization techniques to ensure both data security and the feasibility of model deployment in real-world applications. The results demonstrated the effectiveness of our quantization approach, showing the potential of the framework to automate the analysis of different types of nuclei on various pathological images. This capability not only facilitates faster diagnoses but also enhances our understanding of tissue characteristics, ultimately leading to improved patient care and management. Furthermore, the model's ability to identify and quantify the morphological characteristics of nuclei adds significant diagnostic and predictive value.

Acknowledgements

This work was supported by the National Research Foundation of Korea (NRF) grant funded by the Korean government (MSIT) (No. RS-2023-00218176) and the Soonchunhyang University Research Fund. This grant was approved through J. Kim and Y. Nam, however the funder had no role in writing, decision to publish, or preparation of the manuscript.

Author contributions

Conceptualization: Md Palash Uddin.

Formal analysis: Yunyoung Nam.

Investigation: Jung-Yeon Kim, Yunyoung Nam.

Methodology: Anjir Ahmed Chowdhury, S M Hasan Mahmud, Seifedine Kadry.

Resources: Jung-Yeon Kim.

References

1. Stenkvist B, Westman-Naeser S, Holmquist J, Nordin B, Bengtsson E, Vegelius J, et al. Computerized nuclear morphometry as an objective method for characterizing human cancer cell populations. Cancer Res. 1978;38(12):4688–97. PMID: 82482
2. Elmore JG, Longton GM, Carney PA, Geller BM, Onega T, Tosteson ANA, et al. Diagnostic concordance among pathologists interpreting breast biopsy specimens. JAMA. 2015;313(11):1122–32. https://doi.org/10.1001/jama.2015.1405 PMID: 25781441
3. Spanhol FA, Oliveira LS, Petitjean C, Heutte L. A dataset for breast cancer histopathological image classification. IEEE Trans Biomed Eng. 2016;63(7):1455–62. https://doi.org/10.1109/TBME.2015.2496264 PMID: 26540668
4. Gurcan MN, Boucheron LE, Can A, Madabhushi A, Rajpoot NM, Yener B. Histopathological image analysis: a review. IEEE Rev Biomed Eng. 2009;2:147–71. https://doi.org/10.1109/RBME.2009.2034865 PMID: 20671804
5. Hou L, Gupta R, Van Arnam JS, Zhang Y, Sivalenka K, Samaras D, et al. Dataset of segmented nuclei in hematoxylin and eosin stained histopathology images of ten cancer types. Sci Data. 2020;7(1):185. https://doi.org/10.1038/s41597-020-0528-1 PMID: 32561748
6. Gurcan MN, Tomaszewski JE, Madabhushi A. Special section guest editorial: Digital pathology. 2017.
7. Xie Y, Xing F, Kong X, Su H, Yang L. In: International conference on medical image computing and computer-assisted intervention, 2015. p. 358–65.
8. Zhang Y, Yang L, Chen J, Fredericksen M, Hughes DP, Chen DZ. Deep adversarial networks for biomedical image segmentation utilizing unannotated images. In: International conference on medical image computing and computer-assisted intervention, 2017. p. 408–16.
9. Mahanty C, Kumar R, Asteris PG, Gandomi AH. COVID-19 patient detection based on fusion of transfer learning and fuzzy ensemble models using CXR images. Appl Sci. 2021;11(23):11423. https://doi.org/10.3390/app112311423
10. Collins FS, Varmus H. A new initiative on precision medicine. N Engl J Med. 2015;372(9):793–5. https://doi.org/10.1056/NEJMp1500523 PMID: 25635347
11. Cooper LAD, Kong J, Gutman DA, Wang F, Cholleti SR, Pan TC, et al. An integrative approach for in silico glioma research. IEEE Trans Biomed Eng. 2010;57(10):2617–21. https://doi.org/10.1109/TBME.2010.2060338 PMID: 20656651
12. Aerts HJWL, Velazquez ER, Leijenaar RTH, Parmar C, Grossmann P, Carvalho S, et al. Decoding tumour phenotype by noninvasive imaging using a quantitative radiomics approach. Nat Commun. 2014;5:4006. https://doi.org/10.1038/ncomms5006 PMID: 24892406
13. Altameem A, Mahanty C, Poonia RC, Saudagar AKJ, Kumar R. Breast cancer detection in mammography images using deep convolutional neural networks and fuzzy ensemble modeling techniques. Diagnostics (Basel). 2022;12(8):1812. https://doi.org/10.3390/diagnostics12081812 PMID: 36010164
14. Saeed M, Ahsan M, Saeed MH, Mehmood A, Khalifa HAE-W, Mekawy I. The prognosis of allergy-based diseases using pythagorean fuzzy hypersoft mapping structures and recommending medication. IEEE Access. 2022;10:5681–96. https://doi.org/10.1109/access.2022.3141092
15. Gamper J, Koohbanani NA, Benes K, Graham S, Jahanifar M, Khurram SA. Pannuke dataset extension, insights and baselines. 2020. https://arxiv.org/abs/2003.10778
16. Vuola AO, Akram SU, Kannala J. Mask-RCNN and U-net ensembled for nuclei segmentation. In: 2019 IEEE 16th international symposium on biomedical imaging (ISBI 2019), 2019. p. 208–12.
17. Chen S, Ding C, Tao D. Boundary-assisted region proposal networks for nucleus segmentation. In: International conference on medical image computing and computer-assisted intervention, 2020. p. 279–88.
18. Song Y, Tan E-L, Jiang X, Cheng J-Z, Ni D, Chen S, et al. Accurate cervical cell segmentation from overlapping clumps in pap smear images. IEEE Trans Med Imaging. 2017;36(1):288–300. https://doi.org/10.1109/TMI.2016.2606380 PMID: 27623573
19. Alemi Koohbanani N, Jahanifar M, Gooya A, Rajpoot N. Nuclear instance segmentation using a proposal-free spatially aware deep learning framework. In: International conference on medical image computing and computer-assisted intervention. Springer; 2019. p. 622–30.
20. Chen H, Qi X, Yu L, Heng PA. DCAN: deep contour-aware networks for accurate gland segmentation. In: Proceedings of the IEEE conference on computer vision and pattern recognition, 2016. p. 2487–96.
21. Hwang H, Bui TD, Ahn S i, Shin J. Skipped-hierarchical feature pyramid networks for nuclei instance segmentation. In: 2018 Asia-Pacific signal and information processing association annual summit and conference (APSIPA ASC), 2018. p. 689–93.

22. Ronneberger O, Fischer P, Brox T. U-net: Convolutional networks for biomedical image segmentation. In: International Conference on Medical image computing and computer-assisted intervention, 2015. p. 234–41.
23. Raza SEA, Cheung L, Shaban M, Graham S, Epstein D, Pelengaris S, et al. Micro-Net: A unified model for segmentation of various objects in microscopy images. Med Image Anal. 2019;52:160–73. https://doi.org/10.1016/j.media.2018.12.003 PMID: 30580111
24. Oda H, Roth HR, Chiba K, Sokoli´c J, Kitasaka T, Oda M. BESNet: boundary-enhanced segmentation of cells in histopathological images. In: International conference on medical image computing and computer-assisted intervention, 2018. p. 228–36.
25. Zhou Y, Onder OF, Dou Q, Tsougenis E, Chen H, Heng PA. In: International conference on information processing in medical imaging, 2019. p. 682–93.
26. Gamper J, Alemi Koohbanani N, Benet K, Khuram A, Rajpoot N. Pannuke: an open pan-cancer histology dataset for nuclei instance segmentation and classification. In: European congress on digital pathology, 2019. p. 11–9.
27. Hörst F, Rempe M, Heine L, Seibold C, Keyl J, Baldini G, et al. CellViT: Vision transformers for precise cell segmentation and classification. Med Image Anal. 2024;94:103143. https://doi.org/10.1016/j.media.2024.103143 PMID: 38507894
28. Long J, Shelhamer E, Darrell T. Fully convolutional networks for semantic segmentation. CoRR. 2014. https://doi.org/abs/1411.4038
29. Yu F, Koltun V. Multi-scale context aggregation by dilated convolutions. arXiv Preprint. 2015. https://doi.org/10.48550/arXiv.1511.07122
30. Oktay O, Schlemper J, Folgoc LL, Lee M, Heinrich M, Misawa K. Attention u-net: Learning where to look for the pancreas. 2018. https://arxiv.org/abs/1804.03999
31. Roth HR, Shen C, Oda H, Oda M, Hayashi Y, Misawa K. Deep learning and its application to medical image segmentation. Med Imaging Technol. 2018;36(2):63–71. https://doi.org/10.11409/mit.36.63
32. Badrinarayanan V, Kendall A, Cipolla R. SegNet: A deep convolutional encoder-decoder architecture for image segmentation. IEEE Trans Pattern Anal Mach Intell. 2017;39(12):2481–95. https://doi.org/10.1109/TPAMI.2016.2644615 PMID: 28060704
33. Çiçek O, Abdulkadir A, Lienkamp SS, Brox T, Ronneberger O. 3D U-Net: learning dense volumetric segmentation from sparse annotation. CoRR. 2016. https://doi.org/abs/1606.06650
34. Simonyan K, Zisserman A. Very deep convolutional networks for large-scale image recognition. arXiv Preprint. 2014. https://doi.org/10.48550/arXiv.1409.1556
35. He K, Zhang X, Ren S, Sun J. Deep residual learning for image recognition. In: Proceedings of the IEEE conference on computer vision and pattern recognition, 2016. p. 770–8.
36. Huang G, Liu Z, Van Der Maaten L, Weinberger KQ. Densely connected convolutional networks. In: Proceedings of the IEEE conference on computer vision and pattern recognition, 2017. p. 4700–8.
37. Szegedy C, Liu W, Jia Y, Sermanet P, Reed S, Anguelov D, et al. In: Proceedings of the IEEE conference on computer vision and pattern recognition, 2015. p. 1–9.
38. Li L, Jamieson K, DeSalvo G, Rostamizadeh A, Talwalkar A. Hyperband: A novel bandit-based approach to hyperparameter optimization. J Mach Learn Res. 2017;18(1):6765–816.
39. Jamieson K, Talwalkar A. Non-stochastic best arm identification and hyperparameter optimization. Artificial intelligence and statistics. PMLR. 2016. p. 240–8.
40. Krishnamoorthi R. Quantizing deep convolutional networks for efficient inference: A whitepaper. 2018. https://arxiv.org/abs/1806.08342
41. Grama M, Musat M, Mun oz-Gonz´alez L, Passerat-Palmbach J, Rueckert D, Alansary A. Robust aggregation for adaptive privacy preserving federated learning in healthcare. 2020. https://arxiv.org/abs/2009.08294
42. Li J, Meng Y, Ma L, Du S, Zhu H, Pei Q. A federated learning based privacy-preserving smart healthcare system. IEEE Trans Ind Inform. 2021;18(3).
43. Kairouz P, McMahan HB, Avent B, Bellet A, Bennis M, Nitin Bhagoji A, et al. Advances and open problems in federated learning. FNT Mach Learn. 2021;14(1–2):1–210. https://doi.org/10.1561/2200000083
44. Zhao Y, Li M, Lai L, Suda N, Civin D, Chandra V. Federated learning with non-iid data. 2018. https://arxiv.org/abs/1806.00582
45. Brisimi TS, Chen R, Mela T, Olshevsky A, Paschalidis IC, Shi W. Federated learning of predictive models from federated Electronic Health Records. Int J Med Inform. 2018;112:59–67. https://doi.org/10.1016/j.ijmedinf.2018.01.007 PMID: 29500022
46. Joshi M, Pal A, Sankarasubbu M. Federated learning for healthcare domain-pipeline, applications and challenges. ACM Trans Comput Healthc. 2022.

47. Konečný J, McMahan HB, Yu FX, Richtárik P, Suresh AT, Bacon D. Federated learning: Strategies for improving communication efficiency. 2016. https://arxiv.org/abs/1610.05492

48. Islam TU, Ghasemi R, Mohammed N. Privacy-preserving federated learning model for healthcare data. In: 2022 IEEE 12th annual computing and communication workshop and conference (CCWC), 2022. p. 0281–7. https://doi.org/10.1109/ccwc54503.2022.9720752

49. O'Malley T, Bursztein E, Long J, Chollet F, Jin H, Invernizzi L. Keras Tuner. 2019. https://github.com/keras-team/keras-tuner

50. Tensorflow/federated: A framework for implementing Federated Learning. https://github.com/tensorflow/federated

51. Jetson Nano 2GB Developer Kit - Get Started. https://developer.nvidia.com/embedded/learn/get-started-jetson-nano-2gb-devkit

52. Graham S, Vu QD, Raza SEA, Azam A, Tsang YW, Kwak JT, et al. Hover-Net: Simultaneous segmentation and classification of nuclei in multi-tissue histology images. Med Image Anal. 2019;58:101563. https://doi.org/10.1016/j.media.2019.101563 PMID: 31561183

53. Verma R, Kumar N, Patil A, Kurian NC, Rane S, Graham S, et al. MoNuSAC2020: A multi-organ nuclei segmentation and classification challenge. IEEE Trans Med Imaging. 2021;40(12):3413–23. https://doi.org/10.1109/TMI.2021.3085712 PMID: 34086562

54. Saha M, Chakraborty C. Her2Net: A deep framework for semantic segmentation and classification of cell membranes and nuclei in breast cancer evaluation. IEEE Trans Image Process. 2018;27(5):2189–200. https://doi.org/10.1109/TIP.2018.2795742 PMID: 29432100

55. Ilyas T, Mannan ZI, Khan A, Azam S, Kim H, De Boer F. TSFD-Net: Tissue specific feature distillation network for nuclei segmentation and classification. Neural Netw. 2022;151:1–15. https://doi.org/10.1016/j.neunet.2022.02.020 PMID: 35367734

56. Vu QD, Graham S, Kurc T, To MNN, Shaban M, Qaiser T, et al. Methods for segmentation and classification of digital microscopy tissue images. Front Bioeng Biotechnol. 2019;7:53. https://doi.org/10.3389/fbioe.2019.00053 PMID: 31001524

57. Tommasino C, Russo C, Rinaldi AM, Ciompi F. HoVer-unet: Accelerating hovernet with unet-based multi-class nuclei segmentation via knowledge distillation. In: 2024 IEEE International symposium on biomedical imaging (ISBI). 2024. p. 1–4.
